# Elevated Serum Levels of Soluble TNF Receptors and Adhesion Molecules Are Associated with Diabetic Retinopathy in Patients with Type-1 Diabetes

**DOI:** 10.1155/2015/279393

**Published:** 2015-08-03

**Authors:** Shruti Sharma, Sharad Purohit, Ashok Sharma, Diane Hopkins, Leigh Steed, Bruce Bode, Stephen W. Anderson, Ruth Caldwell, Jin-Xiong She

**Affiliations:** ^1^Center for Biotechnology and Genomic Medicine, Georgia Regents University, 1120 15th Street, Augusta, GA 30912, USA; ^2^Pediatric Endocrine Associates, Atlanta, GA 30342, USA; ^3^Vascular Biology Center, Georgia Regents University, 1120 15th Street, Augusta, GA 30912, USA

## Abstract

*Aims*. To examine the association of the serum levels of TNF receptors, adhesion molecules, and inflammatory mediators with diabetic retinopathy (DR) in T1D patients. *Methods*. Using the multiplex immunoassay, we measured serum levels of eight proteins in 678 T1D subjects aged 20–75 years. Comparisons were made between 482 T1D patients with no complications and 196 T1D patients with DR. *Results*. The levels of sTNFR-I, sTNFR-II, CRP, SAA, sgp130, sIL6R, sVCAM1, and sICAM1 were significantly higher in the T1D patients with DR as compared to T1D patients with no complications. Multivariate logistic regression analysis revealed significant association for five proteins after adjustment for age, sex, and disease duration (sTNFR-I: OR = 1.57, sgp130: OR = 1.43, sVCAM1: OR = 1.27, sICAM1: OR = 1.42, and CRP: OR = 1.15). Conditional logistic regression on matched paired data revealed that subjects in the top quartile for sTNFR-I (OR = 2.13), sTNFR-II (OR = 1.66), sgp130 (OR = 1.82), sIL6R (OR = 1.75), sVCAM1 (OR = 1.98), sICAM1 (OR = 2.23), CRP (OR = 2.40) and SAA (OR = 2.03), had the highest odds of having DR. *Conclusions*. The circulating markers of inflammation, endothelial injury, and TNF signaling are significantly associated with DR in patients with T1D. TNFR-I and TNFR-II receptors are highly correlated, but DR associated more strongly with TNFR-I in these patients.

## 1. Introduction

Diabetic retinopathy (DR) is a sight threatening, microvascular complication of diabetes that affects the retinal vasculature. It is the leading cause of blindness in adults 20–74 years of age in the United States with 28.5% prevalence among 40-year and older patients with diabetes [1]. According to the Centers for Disease Control and Prevention (CDC), the number of Americans aged 40 years and older with DR will triple from 5.5 million in 2005 to 16.0 million in 2050 [2]. So far the only recommended treatment for advanced retinopathy is laser photocoagulation which can control pathological neovascularization but may impair vision and in some patients the retinopathy continues to progress. Clinical trials of anti-VEGF intraocular injections have also shown promise in reducing diabetic macular edema [3]. However, these effects are usually transient and the treatment does not promote tissue repair and the need for repeated injections increases the risk of intraocular infection. Moreover, neither treatment targets early stage disease. Therefore, new markers to define the risk of type-1 diabetes (T1D) associated DR and new therapeutic targets are the critical unmet need.

Several inflammatory proteins are dysregulated in T1D [4] with inflammation being closely associated with the pathogenesis of different complications including DR [5–7]. DR is associated with several microvascular abnormalities such as leukocyte attachment to the vessel walls, occlusion of retinal capillaries, and breakdown of the blood retinal barrier and formation of acellular capillaries. The microvascular injury in DR has been linked to upregulation of several cytokines such as IL-6, IL1-*β*, and VEGF and pathological overexpression of adhesion molecules (ICAM-1 and VCAM-1) [8–10]. The major regulators of vascular adhesion molecules are TNF-*α* and IL-6 [11]. TNF-*α* and IL-6 are pleiotropic cytokines and key molecules in inflammatory signaling with TNF-*α* shown to be involved in the release of IL-6 [12]. IL-6 is known to induce ICAM-1 expression [13], whereas TNF-*α* leads to both ICAM-1 and VCAM-1 expression in endothelial cells [11].

Since adhesion molecules and soluble receptors of TNF-*α* and IL-6 pathway are key mediators of endothelial activation, their elevated levels may represent risk and severity of the pathogenesis of DR. Therefore, we examined the levels of soluble TNF receptors (sTNFR-I and sTNFR-II), soluble IL-6 receptors (sIL6R, sgp130), adhesion molecules (sICAM-1, sVCAM-1), and inflammatory markers (CRP, SAA) in serum of T1D patients with and without DR. The aim of this study was to examine the association of the serum levels of these inflammatory mediators with DR and to determine if these markers could be used as surrogate endpoints to define the risk of DR in T1D patients.

## 2. Research Design and Methods

### 2.1. Human Subjects and Serum Samples

This study was approved by the institutional review board of the Georgia Regents University, Augusta, Georgia. Blood samples from the participants of Phenome and Genome of Diabetes Autoimmunity (PAGODA) study were obtained after the informed consent from the subjects. All subjects were recruited in the state of Georgia, USA, mainly in the Atlanta and Augusta city areas. The demographic information for T1D subjects with no complications and with DR is presented in Table 1.

Peripheral blood was collected in serum separator tubes (BD Biosciences, San Jose, CA, USA) and clotted for 30 minutes, the tubes were centrifuged, and serum was immediately aliquoted and stored in −80°C freezers. Serum samples from T1D patients were aliquoted randomly into 96 well plates and each plate contained similar numbers of samples from T1D patients with and without DR.

### 2.2. Luminex Immunoassays

Luminex immunoassays for sTNFR-I, sTNFR-II, CRP, SAA, sIL6R, sgp130, sICAM-1, and sVCAM1 were obtained from Millipore (Millipore Inc., Billerica, MA, USA). Multiplex immunoassays were performed according to the manufacturer's instructions. Briefly, serum samples were incubated with antibody-coated microspheres, followed by biotinylated detection antibody. Proteins were detected by incubation with phycoerythrin-labeled streptavidin and the resultant bead immunocomplexes were read on a FLEXMAP3D (Luminex, TX, USA) with the following instrument settings: events/bead: 50, minimum events: 0, flow rate: 60 *μ*L/min, sample size: 50 *μ*L, and discriminator gate: 8000–13500. Median fluorescence intensity (MFI) was collected and used for calculating protein concentration.

### 2.3. Statistical Analyses

All statistical analyses were performed using the R language and environment for statistical computing (R version 2.15.1; R Foundation for Statistical Computing; http://www.r-project.org/). All *P* values were two-tailed and a *P* < 0.05 was considered statistically significant.

Protein concentrations were estimated using a regression fit to the standard curve with known concentration included on each plate using a serial dilution series. To achieve normal distribution, the concentrations were log2 transformed prior to all statistical analyses. The potential differences between T1D patients without any complication and T1D patients with retinopathy were initially examined using a *t*-test. The pairwise correlation between individual protein levels was computed using Pearson correlation coefficient. Clustering and visualization of correlation matrix was performed using hierarchical clustering method and heatmap. The effect of age and T1D duration on serum levels of each candidate molecule was determined using a linear regression by including age or T1D duration as covariate on data stratified by sex and disease status. To examine the relationships between retinopathy and the serum protein levels logistic regression was used. Age, sex, and T1D duration were included as covariates in a stepwise manner.

To estimate the risk of diabetes at different protein concentrations, we performed conditional logistic regression on matched paired data. Case-control matching was performed with respect to age, sex, and T1D duration using the “matching” R package [14]. The odds ratios and 95% confidence intervals (CI) were computed for each protein and protein concentration was used as categorical variable (values 1, 2, 3, and 4 were assigned using the quartile values in controls as cutoff points).

## 3. Results

Serum levels of eight proteins in 482 T1D patients with no complications and 196 T1D patients with DR were measured. The demographic information and baseline characteristics of the subjects involved in this study are shown in Table 1. The average age of the T1D subjects without any complications was 39.1 ± 12.7 years and for subjects with DR was 49.3 ± 11.6. The duration of diabetes in patients without complications was 18.2 ± 11.1 as compared to 31.4 ± 10.3 in patients with DR.

### 3.1. Alterations in Serum Protein Levels in T1D Patients with DR

**Figure 1:**
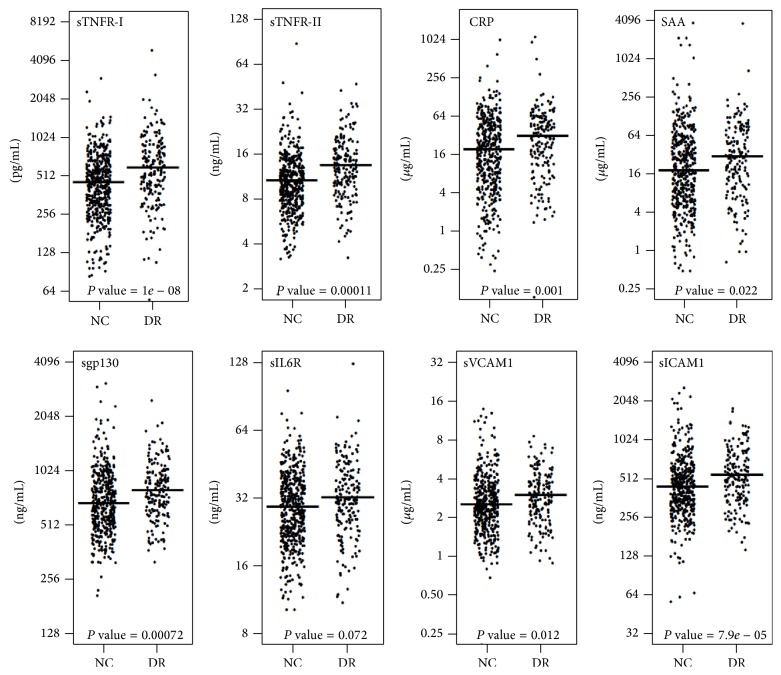
Elevated serum protein levels in T1D subjects with DR. Serum levels of eight proteins were measured in 678 T1D subjects aged 20–75 years. Comparisons were made between 482 T1D patients with no complications and 196 T1D patients with diabetic retinopathy. Plots depict the distribution of the protein levels in two different groups.

**Figure 2:**
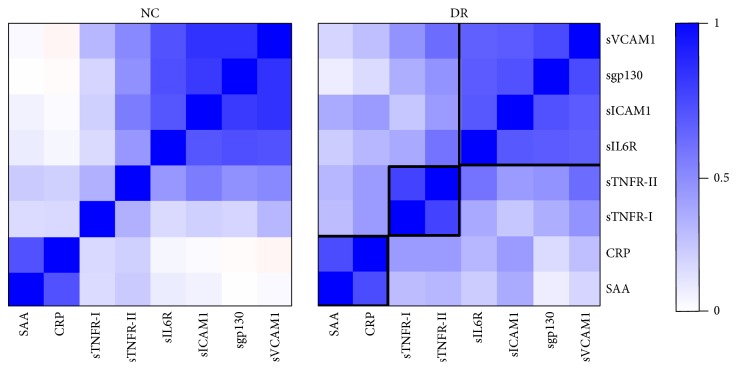
Three clusters of functionally related proteins with strong positive correlations. The pairwise correlations between all eight proteins were examined in T1D patients with and without DR separately. Correlation between individual protein levels was computed using Pearson correlation coefficient. Clustering and visualization of correlation matrix was performed using hierarchical clustering method and heatmap. Three clusters of functionally related proteins were found with strong positive correlations.

The levels of all eight molecules were significantly higher in the T1D patients with DR as compared to T1D patients with no complications: sTNFR-I (1.30-fold), sTNFR-II (1.27-fold), CRP (1.53-fold), SAA (1.33-fold), sgp130 (1.14-fold), sIL6R (1.08-fold), sVCAM1 (1.11-fold), and sICAM1 (1.19-fold) as shown in Figure 1. Next, we examined the pairwise correlations between all eight proteins and hierarchical clustering of the correlation matrix was performed in T1D patients with and without DR separately (Figure 2). We found three clusters of functionally related proteins with strong positive correlations. The proteins in cluster-1 include sgp130, sVCAM1, sICAM1, and sIL6R and the proteins in cluster-2 are CRP and SAA. The third cluster of proteins with strong positive correlation includes sTNFR-I and sTNFR-II. The correlations were almost similar in both no complication and DR groups (Figure 2) except sTNFR-II. The correlation of sTNFR-II was increased with other proteins in DR group as compared to the T1D group without any complications.

### 3.2. Multivariate Logistic Regression Analysis Reveals Significant Association between Protein Levels and DR

Multivariate logistic regression analysis was performed using four different models (Model 1: no adjustments, model 2: adjusted for age, model 3: adjusted for age and sex, and model 4: adjusted for age, sex, and T1D duration). The odds ratio of five proteins showed significant association with diabetic retinopathy (Table 2). We found that three proteins directly involved in TNF/IL-6-pathway have larger odds ratios (sTNFR-I: OR = 1.57, sICAM1: OR = 1.42, and sgp130: OR = 1.43). All eight proteins have positive associations with DR for the four models (Table 2).

### 3.3. Risk for DR Is Directly Related to the Protein Levels

**Figure 3:**
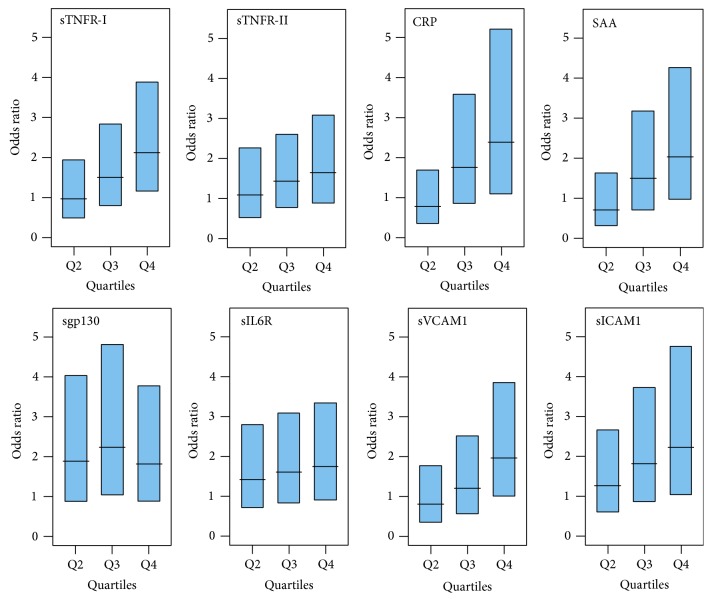
Strong association of increasing protein levels with DR. Conditional logistic regression was performed on matched paired data using cases (T1D with DR) and controls (T1D without complication) matched for age, sex, and T1D duration (183 pairs). Subjects were divided into four quartiles based on individual protein levels. The odds ratios and 95% confidence intervals (CI) were computed for each protein using lowest quartile as referent. Compared with subjects in the bottom quartile, subjects in the top quartile had the highest risk of DR for all eight proteins. Also, an increased trend in the risk for DR was observed from quartile-2 to quartile-4 of protein concentrations.

Since there was a significant effect of age, sex, and T1D duration on the protein concentrations, a paired dataset of 183 matched pairs was generated using multivariate and propensity score matching software [14]. Matching was performed with respect to age, sex, and duration of diabetes and each T1D patient with DR was paired with closest T1D patient without complication. The demographic information and baseline characteristics of the samples after matching are presented in Table 3. Conditional logistic regression was performed to estimate the risk of DR at different protein concentrations. Protein levels were used as categorical variable after dividing into 4 quartiles. The odds ratios of having DR were computed for quartile-2, quartile-3, and quartile-4 using quartile-1 as reference. Subjects in the top quartile had the highest risk of DR compared with subjects in the bottom quartile for all eight proteins: sTNFR-I (OR = 2.13), sTNFR-II (OR = 1.66), CRP (OR = 2.40), SAA (OR = 2.03), sgp130 (OR = 1.83), sIL6R (OR = 1.75), sVCAM1 (OR = 1.98), and sICAM1 (OR = 2.23). Also, for all proteins, an increased trend in the risk for DR was observed from quartile-2 to quartile-4 of protein concentrations as shown in Figure 3.

## 4. Conclusions

Hyperglycemia and aging activate multiple cellular pathways which play an important role in diabetic retinopathy. Previous studies have related inflammation and endothelial injury to be closely associated with the pathogenesis of microvascular complications including DR [5–7]. In this study, we measured serum levels of 8 proteins in blood samples from T1D patients with DR and T1D patients without any complications. We found significant alterations in the serum protein levels of sTNFR-I, sTNFR-II, CRP, SAA, sIL6R, sgp130, sVCAM1, and sICAM1.

TNF-*α* has been shown to be involved in the development and progression of DR [15]. Studies have shown the importance of TNF-*α* system in diabetic retinal microvascular damage [16]. TNF-*α* binds to its membrane receptors, TNFR-I and TNFR-II, which initiate signaling pathway leading to activation of transcription factors such as NF-*κ*B as well as apoptosis [17]. In animal models, drugs that target TNF-*α* have been shown to reduce leukostasis, retinal vascular leakage, and retinal cell death [18, 19]. Proteolytic cleavage of extracellular domains of TNF-*α* receptors results in their release as soluble forms (sTNFR-I and sTNFR-II). While these 2 receptors are well-known as TNF antagonist, these can also act as a reservoir of circulating TNF-*α*. Recent studies have shown that these soluble forms may be more important than TNF-*α* itself in regulation of TNF signaling [20]. We found that sTNFR-I and sTNFR-II both are upregulated in T1D patients with DR. sTNFR-I and sTNFR-II receptors were highly correlated, but DR associated more strongly with sTNFR-I in these patients.

Earlier studies have also reported that the serum and vitreous levels of sTNFRs are elevated in DR patients [21], TNFR-I expression may be a more significant target than TNF-*α* for intervention in ocular inflammation [20], and TNF-*α* inhibition is known to reduce the leukocyte adhesion in the retina and the loss of retinal microvascular cells in diabetic rats. Also, activated TNF-*α* might regulate blood-retinal barrier (BRB) breakdown, retinal leukostasis, and apoptosis in later stages of DR [22]. Thus, the effective control of TNF-*α* activity by sTNFRs within the retinal microenvironment may determine the outcome and severity of DR.

Interestingly, we found significant alterations in soluble glycoprotein 130 (sgp130) protein levels that has not been previously implicated in DR. This protein plays a crucial role in IL-6 trans-signaling. Increasing evidences suggest that IL-6 pathway plays a prominent role in the pathogenesis of DR and IL-6 and its soluble receptor (sIL-6R) operate as central regulators of the inflammatory processes [23, 24]. The effect of IL-6 on target cells is mediated by a complex receptor system, composed of IL-6R (gp80) and a signal-transducing glycoprotein (gp130) [25]. IL-6 signals to target cells by binding to the cell-surface IL-6R receptors known as “classic” signaling pathway. On the other hand, IL-6/sIL-6R complex can also bind to cell-surface glycoprotein 130 (gp130) on cells which do not express the IL-6R. This process has been called “IL-6 trans-signaling” mediated by gp130. The recent findings implicate IL-6 trans-signaling in inflammation and related diseases in humans and mice [26–28]. In animal models of inflammation it has been shown that sgp130 administration decreases disease severity [29–31]. Elevated sgp130 serum concentrations were found in inflammatory diseases, such as Crohn's disease, rheumatoid arthritis, or inflammatory colon cancer [32–34]. Further studies will be required to elucidate the mechanisms of sgp130 regulation and the implications of targeting it as a therapeutic agent in DR.

The rapid, massive shedding of membrane-bound ICAM-1 from EC leads to an increase in circulating soluble ICAM-1 concentration which has been reported as a biomarker for inflammation and EC activation [35]. We measured these markers of endothelial activation and found them to be higher in T1D patients with DR. These molecules are expressed on the endothelial cell surface and their increased production results in recruitment and activation of granulocytes, monocytes/macrophages, and lymphocytes at the damaged tissue site [36]. These selectins also mediate initial rolling of leukocytes along the endothelium and play important roles in the firm attachment and transendothelial migration of leukocytes. TNF-*α* has been shown to induce expression of both ICAM and VCAM in endothelial cells [11]. Thus, our study suggests that serum sICAM1 and sVCAM1 concentrations may reflect TNF-*α* mediated progression and severity of DR associated with T1D.

Studies have also shown the association of inflammatory biomarkers such as CRP and SAA with T1D and other microvascular complications [37–40]. CRP induces proinflammatory effects through overproduction of ICAM-1 and VCAM-1 adhesion molecules. Also, IL-6 activation leads to production of CRP which has been previously shown to be elevated in adults with T1D [37, 41]. In the Diabetes Autoimmunity Study of the Young (DAISY), elevated CRP levels were more frequent in children who later developed T1D and provide evidence that the disease is an immunoinflammatory disorder [37]. CRP is shown to mediate endothelial dysfunction by inhibiting endothelium-dependent NO-mediated dilation in retinal arterioles by producing superoxide from NADPH oxidase [42]. Recently, a study in a rat model has shown that elevated CRP levels are associated with increased cardiovascular events and endothelial dysfunction [43]. Another study has reported that elevated CRP levels in T1D patients were not associated with glycemic control but reflected a low-grade inflammation associated with the activation of innate immune activity [37]. In our study, circulating levels of CRP and SAA (a similar inflammatory marker) were increased in T1D subjects with DR as compared to T1D patients with no complications.

In conclusion, this study reveals that serum levels of TNF receptors, adhesion molecules, and other inflammatory mediators could be used as surrogate endpoints in studies of interventions to decrease inflammation among subjects with T1D. Significant associations between systemic markers of inflammation highlight that subclinical inflammation might be a mechanism through which hyperglycemia causes DR with endothelial impairment playing an important role in the pathogenesis of DR. However, future studies will be required to determine the precise understanding of whether these elevated biomarkers are participating in or are an indicator of DR progression.

## Figures and Tables

**Table 1:** Baseline characteristics of T1D subjects without complications and with diabetic retinopathy (DR).

Patient characteristics	Without any complications	With DR	*P* value
Subjects (*n*)	482	196	—
Female (%)	52.1	61.7	0.027
Age (years)	39.1 ± 12.7	49.3 ± 11.6	1.09*E* − 21
Age range (years)	20.0 to 73.8	24.6 to 73.8	—
Duration of disease	18.2 ± 11.1	31.4 ± 10.3	1.09*E* − 21
Systolic BP (mmHg)	117.8 ± 9.5	122.9 ± 12.1	1.17*E* − 06
Diastolic BP (mmHg)	73.7 ± 6.4	73.8 ± 7.3	0.208
Diabetic nephropathy (%)	0	0	—
Diabetic neuropathy (%)	0	41.8 (*n* = 82)	—
CAD (%)	0	16.3 (*n* = 32)	—
Dyslipidemia (N/Y)	376/106	112/84	7.05*E* − 08
Hypertension (N/Y)	425/57	117/79	1.14*E* − 16
Hemoglobin	14.4 ± 1.5	13.7 ± 1.6	1.40*E* − 04
Albumin	4.4 ± 0.4	4.2 ± 0.4	1.79*E* − 05
LDL	94.0 ± 27.4	102.4 ± 38.3	0.693
Total cholesterol	174.6 ± 34.2	184.1 ± 47.1	0.565
Triglycerides	90.4 ± 67.6	109.5 ± 94.2	0.395
HDL	61.9 ± 17.9	60.7 ± 19.4	0.639
Creatinine	0.9 ± 0.2	1.0 ± 0.2	7.0*E* − 04
HbA1c	7.8 ± 1.1	8.0 ± 1.3	0.319
BUN	13.4 ± 4.5	16.4 ± 5.6	4.83*E* − 08
Microalbumin	24.9 ± 122.3	85.2 ± 252.1	0.017

**Table 2:** Multivariate logistic regression analysis for proteins after adjustment for age, sex, and disease duration.

	Model 1	Model 2	Model 3	Model 4
	OR (CI 95%)	OR (CI 95%)	OR (CI 95%)	OR (CI 95%)
	*P* value	*P* value	*P* value	*P* value
sTNFR-I	1.83 (1.49–2.28)	1.62 (1.31–2.02)	1.67 (1.35–2.09)	1.57 (1.25–2.00)
	2.67 × 10^−8^	3.24 × 10^−9^	4.00 × 10^−6^	1.66 × 10^−4^
sTNFR-II	1.54 (1.24–1.97)	1.32 (1.09–1.64)	1.32 (1.09–1.64)	1.18 (0.97–1.46)
	2.46 × 10^−4^	7.44 × 10^−3^	7.67 × 10^−3^	0.113
CRP	1.16 (1.06–1.27)	1.16 (1.06–1.28)	1.16 (1.05–1.28)	1.15 (1.04–1.29)
	1.51 × 10^−3^	2.68 × 10^−3^	4.07 × 10^−3^	9.37 × 10^−3^
SAA	1.10 (1.01–1.19)	1.07 (0.98–1.16)	1.06 (0.97–1.16)	1.10 (1.00–1.22)
	0.025	0.145	0.200	0.046
sgp130	1.59 (1.21–2.14)	1.58 (1.18–2.15)	1.58 (1.19–2.16)	1.43 (1.05–1.97)
	1.61 × 10^−3^	2.71 × 10^−3^	2.71 × 10^−3^	0.026
sIL6R	1.26 (1.00–1.63)	1.27 (0.99–1.69)	1.28 (1.00–1.70)	1.25 (0.96–1.66)
	0.069	0.077	0.070	0.108
sVCAM1	1.30 (1.05–1.64)	1.28 (1.01–1.63)	1.28 (1.01–1.63)	1.27 (0.98–1.65)
	0.021	0.044	0.046	0.074
sICAM1	1.59 (1.25–2.04)	1.56 (1.20–2.03)	1.54 (1.19–2.01)	1.42 (1.07–1.89)
	1.65 × 10^−4^	8.6 × 10^−4^	1.08 × 10^−3^	0.015

Model 1: no adjustments, model 2: adjusted for age, model 3: adjusted for age and sex, and model 4: adjusted for age, sex, and T1D duration.

**Table 3:** Baseline characteristics of T1D subjects without complications and with diabetic retinopathy after matching.

Patient characteristics	Without any complications	With diabetic retinopathy
Subjects (*n*)	183	183
Female (%)	37.7	37.7
Age (years)	48.15 ± 10.7	48.9 ± 11.5
Age range (years)	21.7 to 70.5	24.6 to 73.8
Duration of disease	30.7 ± 10.7	30.9 ± 10.3
Diagnosis age	17.8 ± 12.4	18.2 ± 12.5
Systolic BP (mmHg)	120.7 ± 10.4	124.1 ± 12.2
Diastolic BP (mmHg)	72.3 ± 6.2	72.7 ± 8.3
Diabetic nephropathy (%)	0	0
Diabetic neuropathy (%)	0	41.0 (*n* = 75)
CAD (%)	0	13.6 (*n* = 25)
